# Fermented *Astragalus* in diet altered the composition of fecal microbiota in broiler chickens

**DOI:** 10.1186/s13568-018-0682-4

**Published:** 2018-09-25

**Authors:** Hongxing Qiao, Yuzhen Song, Hongtao Shi, Chuanzhou Bian

**Affiliations:** 1 College of Veterinary Medicine, Henan University of Animal Husbandry and Economy, Longzihu North road NO. 6, Zhengzhou, Henan People’s Republic of China; 2 Probiotics Bio-transformation Engineering Technology Research Center of Henan Province, Longzihu North road NO. 6, Zhengzhou, Henan People’s Republic of China; 3 Key Laboratory of Probiotics Fermentation Traditional Chinese Medicine of Zhengzhou city, Longzihu North road NO. 6, Zhengzhou, 450046 Henan People’s Republic of China

**Keywords:** Fermented *Astragalus*, Growth performance, Serum biochemical parameters, Microbiota, Broiler chickens, 16S rRNA sequencing

## Abstract

The composition and function of the intestinal microbiota play important roles in digestion and degradation of herbal medicines (HMs). However, few studies have examined the relationship between the fecal microbiota and HMs. In this study the effect of unfermented *Astragalus* (UA) and fermented *Astragalus* (FA) on growth performance, serum biochemical parameters, and fecal microbiota was evaluated in broiler chickens. In total, 180 one-day-old broiler chickens (Avian breeds) were randomly assigned to a control (C) group fed a basal diet, an unfermented (U) group fed a basal diet containing 0.5% UA, or a fermented (F) group fed a basal diet containing 0.5% FA, for 42 days. The F/G ratio was lower in F and U groups than in C group from 22 to 42 days (*P* < 0.05). Glutathione superoxide dismutase, antioxidant capacity, and total superoxide dismutase were higher, whereas malondialdehyde was lower in F group than in C and U groups from 1 to 21 days and from 22 to 42 days (*P* < 0.05). Fecal microbiota were profiled on an Illumina MiSeq platform following PCR amplification of the V4 region of the 16S rRNA gene. At the genus level *Lactobacillus* was the most abundant genus on day 7 in F group. Importantly, a potentially pathogenic genus, *Enterococcus,* was less abundant in the U and F groups than in the C group on day 35 (*P* < 0.05). These results indicate that dietary supplementation with 0.5% FA has beneficial effects on growth performance, serum biochemical parameters and fecal microbiota of broiler chickens.

## Introduction

Over the past decades, the application of antibiotics has improved growth rate and feed conversion efficiency in poultry production (Sugiharto 2014). However, increased use of antibiotics in livestock and poultry production has prompted widespread concern about the prevalence of antibiotic-resistant bacteria. In particular, the accumulation of antibiotic residue in chickens results in the contamination of meat and eggs (Suresh et al. 2018). Increasing consumer awareness of food safety and biosecurity concerns is driving the poultry industry to look for alternatives to feed additives (Yitbarek et al. 2015). Previous studies have shown that herbal feed additives promote growth performance, improve immune function, while exerting anti-bacterial, anti-viral, and anti-oxidative effects in livestock (Wang et al. 2014b; Hu et al. 2011).

The root of *Astragalus membranaceus* (Fisch.) Bge. var*. mongholicus,* a common herbal medicine, contains polysaccharides, saponins, flavonoids, anthraquinones, alkaloids, amino acids, β-sitosterol, and metallic elements and has long been used as a feed additive in the livestock and poultry industries in China (Ibrahim et al. 2013; Li et al. 2009). The major polysaccharides present in *Astragalus* are mannose, d-glucose, d-galactose, xylose, and l-arabinose (Kallon et al. 2013). The major flavonoids of *Astragalus* are 3-O-β-d-glucoside, 2′-hydroxy-3′,4′-dimethoxyisoflavane-7-O-β-d-glycoside, 7,3′-dihydroxy-4′-methoxyisoflavone, 7,3-dimer-capto-4,1-methoxyisoflavone, 3-dimercapto-7,4,1-methoxyisoflavone, and kumatakenin (Lv et al. 2011; Xiao et al. 2004). A recent study showed that crude extracts of *Astragalus* are anti-inflammatory (Kim et al. 2013), anti-viral (Zhang et al. 2018), immunostimulatory (Zhang et al. 2017), promote growth performance (Li et al. 2018) and affects fecal microbiota of young hens (Qiao et al. 2018a). In recent years, fermentation has become a powerful tool for producing biological materials, degrading macromolecular material into small molecules, reducing the side effects associated with herbs such as *Aristolochia* plants, and introducing medicinal effects through biological modification (Lin and Chiang. 2008; Zhou et al. 2015; Luciano and Perazella 2015). Chinese herbs have long been processed using microbial fermentation. Solid state fermentation (SSF) is known to reduce anti-nutritional factors and improve the nutritional quality of feedstuffs (Ahmed et al. 2017). The fermentation of *Astragalus* by *Aspergillus oryzae* M29 improves antioxidant activity and increases phenolic content (Sheih et al. 2011). We previously showed that *Astragalus* fermentation by *Bacillus subtilis* using liquid fermentation technology promotes *Astragalus* polysaccharide production (Qiao et al. 2017).

Many herbal supplements include carbohydrates, particularly polysaccharides and oligosaccharides, which promote pharmacological immunomodulatory, antitumor, antioxidant, hypoglycemic, and anti-inflammatory effects (Li et al. 2013). However, most herbal carbohydrates can not be digested and used by the host in the absence of microbial fermentation (Vrize et al. 2010). Polysaccharides fermented by intestinal microbiota can be converted into short-chain fatty acids (SCFAs), which are beneficial to the host (Lyu et al. 2017). Similarly, flavonoids are important polyphenolic compounds in these herbs that may be more easily absorbed in the intestines after fermentation (Izumi et al. 2000). Supplementation with fermented herbs can trigger secretion of digestive enzymes, enhance digestion of nutrients in the intestine (Yu et al. 2015), modulate the gut microbiota and increase the proportion of beneficial microbiota (Zhang et al. 2015).

In previous studies, fermented herbs, including *Ginkgo biloba* leaves, *Pinus* needles, reportedly improve growth performance, feed efficiency, meat quality, immune function, and blood parameters in livestock (Ahmed et al. 2016; Wu et al. 2015; Zhang et al. 2015). Fermented herbs such as *Anoectochilus formosanus* Hyata, *Flos Lonicera*, and *Rhizoma Atractylodis Macrocephalae* have antioxidant activity and modulate the intestinal microbiota of rats (Ng et al. 2011; Wang et al. 2014a, 2015). *Astragalus membranaceus* root improves growth performance, antioxidant status, and serum metabolites of broilers chickens (Zhang et al. 2013). Although *Astragalus* fermentation has been studied in the past, the effects of fermented *Astragalus* (FA) in broiler chickens have not been investigated.

In this study, we determine the effects of *Astragalus* fermented by *Lactobacillus plantarum* on growth performance, blood characteristics, and fecal microbiota of broiler chickens.

## Materials and methods

### Fermentation of *Astragalus*

The dried root of *Astragalus membranaceus* (Fisch.) Bge. var*. mongholicus* was obtained from Gansu Huisen Pharmaceutical Development Co., Ltd. (Minxian, Gansu, China) and verified by Dr. JingYu Zhang (Henan University of Traditional Chinese Medicine, Zhengzhou, Henan, China). The fermentation of *Astragalus* was performed following our laboratory-optimized procedure as follows: Briefly, *Astragalus* was ground into a powder using a 100-mesh screen. The dried powder (2500 g) was inoculated with 10^5^ colony-forming units (CFU)/mL of *L. plantarum* (CGMCC 1.557), which was originally obtained from the China General Microbiological Culture Collection Center (CGMCC). We previously reported the production parameters for fermenting *Astragalus* with *L. plantarum*: acetic acid, methylacetic acid, aethyl acetic acid and lactic acid were 1723.01 mg/kg, 95.34 mg/kg, 397.22 mg/kg, and 1946.17 mg/kg on day 15, respectively. Other production were: polysaccharides 9.43% on day 10; saponins, 20.1761 mg/g on day 25, flavonoids 1.9153 mg/g on day 15 (Qiao et al. 2018b). The *L. plantarum* strain was cultured in de Man, Rogosa, and Sharpe (MRS) medium at 37 °C for 18 h. Fermentation was conducted in 35 × 45-mm plastic film bags (Zhejiang Jinhu Plastics Machinery Co., Zhejiang, China), which were vacuum-sealed. The mixture was then incubated for 96 h at 37 °C under anaerobic conditions. At the end of the fermentation process, optimal fermentation was confirmed by calculating the number of *L. plantarum* CFUs, and measuring the pH of the FA preparation (the pH of the fermented product should be between 4.0 and 4.5). Unfermented *Astragalus* (UA) was ground into a powder using a 100-mesh screen, inoculated into MRS medium without *L. plantarum*, and cultured for 96 h at 37 °C under anaerobic conditions. FA and UA samples were spread on a polythene sheet and dried for 4 days up to 90% of its dry weight at 30–40 °C, and then ground. The results of nutritional analyses of *Astragalus* before and after fermentation are shown in Table 1.

**Table 1:** Nutritional composition of *Astragalus* before and after fermentation

Items	Before fermentation	After fermentation
Polysaccharides (%)	4.21	7.43
Total flavonoids (mg/g)	28.25	26.15
Total saponins (mg/g)	4.0	5.0
Ferment cells number (CFU/g)	0	9.2 × 10^10^
Acetic acid (mg/kg)	0	421.14
Methylacetic acid (mg/kg)	0	805.29
Ethyl acetic acid (mg/kg)	0	31.25
Lactic acid (mg/kg)	0	92.44

### Experimental design and broiler chicken housing

A total of 180 one-day-old broiler chickens (Avian breeds) with average initial body weight (BW) of 51.45 g were purchased from a local commercial hatchery (Xinzheng, Henan, China). Birds were randomly assigned to one of three groups comprising six pens, with each pen containing ten birds (50% male and 50% female) and provided with feed and water ad libitum according to their BW during the 45-day experimental period. Each group was housed in an individual house of > 30 m^2^ to avoid between-group interference. Broilers and environmental parameters were checked twice daily by trained staff. From day 1 to 21, the temperature and humidity were maintained at 33–35 °C and 60–70%, respectively. From day 22 to 42, the temperature and humidity were maintained at 24–28 °C and 50–60%, respectively. All procedures were performed in accordance with the guidelines of the Chinese Agricultural Ministry. Basal diets were administered as mash and nutrient levels for the starter phase from day 1 to 21 and grower phase from day 22 to 42 were formulated to meet the Feeding Standard of Chicken of the People’s Republic of China (NY/T 33–2004). One group served as the control (C) group and was fed only conventional feed, while the U and F groups were fed feed mixed with either UA (0.5%) or FA (0.5%), respectively, throughout the entire experimental period (Table 2). All animal experiments were conducted according to the Guidelines for the Care and Use of Experimental Animals established and approved by the Laboratory Animal Management Committee of Henan University of Animal Husbandry and Economy (HNMY 1606).

**Table 2:** Composition of the basic diet (as a percentage of dried weight)

Ingredient	1–21 days			22–45 days		
	C group	U group	F group	C group	U group	F group
Corn	60.35	59.85	59.85	62.55	62.05	62.05
Soybean meal	28.25	28.25	28.25	26.15	26.15	26.15
Flour	4.0	4.0	4.0	5.0	5.0	5.0
Corn gluten meal	3.0	3.0	3.0	2.5	2.5	2.5
Fishmeal	1.2	1.2	1.2	1.0	1.0	1.0
Dicalcium phosphate	1.4	1.4	1.4	1.2	1.2	1.2
Limestone	1.2	1.2	1.2	1.0	1.0	1.0
NaCl	0.3	0.3	0.3	0.3	0.3	0.3
Oil	0.3	0.3	0.3	0.3	0.3	0.3
Fermented *Astraglus*	0	0	0.5	0	0	0.5
Unfermented *Astraglus*	0	0.5	0	0	0.5	0
Total	100	100	100	100	100	100

*C group* basal diet,* U group* basal diet + 0.5% unfermented *Astragalus, F group* basal diet + 0.5% fermented*Astragalus*

### Sample collection and measurements

The body weight of individual broiler chickens (on days 1, 21, and 42, weighed in the morning before feeding) and the amount of feed consumed by each group were recorded weekly and used to calculate the average daily feed intake (ADFI), average daily gain (ADG), and feed to gain (F/G) ratio per cage. The ADFI, ADG, and F/G ratio were determined separately for the starter (1–21 days) and grower (22–42 days) phases.

Six broiler chickens from each treatment group were selected randomly in the morning of day 21 and 42 of the experiment and feed was removed for 12 h. The BW of each bird was measured after which blood samples (5.0 mL) were taken from the wing vein. Blood samples were incubated at 37 °C for 30 min and subsequently separated by centrifugation at 12,000×*g* for 10 min at 4 °C. Serum samples were frozen at − 20 °C prior to serum biochemical parameter measurements. Levels of serum catalase (CAT), glutathione superoxide dismutase (GSH-Px), malondialdehyde (MDA), superoxide dismutase (SOD), and antioxidant capacity (AOC) were analyzed by spectrophotometrical methods with ELISA assay kits (Nanjing Jiancheng Bioengineering Institute, Nanjing, China) according to the manufacturer’s instructions. These antioxidant enzymes play an important role in balancing redox status (Wu et al. 2015).

Forty-five fecal samples were chosen randomly (five samples per group on days 7, 21, and 35, respectively) and stored immediately at − 20 °C prior to DNA extraction. DNA was isolated from 200 mg of feces from each bird using a DNA isolation kit (TIANGEN, cat#DP328) according to the manufacturer’s instructions. The quality of the extracted DNA was assessed by 0.8% agarose gel electrophoresis and spectrophotometry (optical density at 260/280 nm). The extracted DNA was stored at − 20 °C until further analysis.

For library preparation, the V4 region of the 16S rRNA gene was amplified from 10 ng of DNA from each fecal sample. The V4 region of the bacterial 16S rRNA gene was amplified by polymerase chain reaction (PCR) (98 °C for 30 s, followed by 27 cycles at 98 °C for 15 s, 50 °C for 30 s, and 72 °C for 30 s, followed by a final extension at 72 °C for 5 min) using the primers 5′-barcode–AYT GGG YDT AAA GNG–3′ and 5′–TAC NVG GGT ATC TAA TCC–3′, where the bar code was a seven-base sequence unique to each sample. PCRs were performed in triplicate in a 20-μL mixture containing 5 μL of 5 × reaction buffer, 5 × High GC buffer, 0.25 μL of Q5 high-fidelity DNA polymerase, 1 μL of each primer (10 μM), 0.5 μL of dNTPs (10 μM), 1 μL of template DNA, and 11.25 μL of distilled water. The amplicons were extracted from 2% agarose gels and then purified using the Axygen^®^ AP-GX-250 AxyPrep™ DNA Gel Extraction Kit (Corning Life Sciences, Corning, NY, USA) according to the manufacturer’s instructions. The library was prepared using the TruSeq Nano DNA LT Library Prep Kit (Illumina, San Diego, CA, USA) and quantified using the Quant-iT PicoGreen dsDNA Assay Kit (Invitrogen Corporation, Carlsbad, CA, USA). The qualified library was subsequently diluted to 2 nM before sequencing.

For the library, paired-end sequencing (2 × 300 bp) was performed on an Illumina MiSeq platform using the MiSeq Reagent Kit V3 (600-cycles-PE) (MS-102-3033). A pooled library (2 nM) was mixed with fresh 0.1 N NaOH and then sequenced. The molarity of the library was controlled to within 15 pM. Library preparation and sequencing was performed by Personal Biotechnology Co., Ltd. (Shanghai, China).

Raw reads were quality filtered to remove chimeric reads, reads < 150 bp, or with average phred scores < 20. The remaining high-quality reads were processed using the Quantitative Insights into Microbial Ecology (QIIME) package v1.8 (Caporaso et al. 2010), merged and clustered into operational taxonomic units (OTUs) based on a 97% sequence similarity threshold. Taxonomic assignment was performed against the Greengenes reference database [the Ribosomal Database Project (RDP) Gold database, 2.2]. Next, archaeal and eukaryotic reads were removed to minimize the effect of very low abundance OTUs (Bokulich et al. 2013). Alpha diversity was estimated using the Chao 1, ACE, Shannon and Simpson indices. Beta diversity was determined by principal coordinate analyses (PCoA) (Lozupone and Knight 2005) using weighted and unweighted UniFrac distance metrics. While both weighted and unweighted UniFrac take into account phylogenetic distances between OTUs, unweighted UniFrac distance is a qualitative measure and does not take into account OTU abundance, and only considers presence/absence (Lozupone and Knight 2005), while weighted UniFrac is a quantitative measure that takes into account OTU abundance (Lozupone et al. 2007). Raw sequence reads were deposited to the National Center for Biotechnology Information Short Read Archive (SRA) under the accession number SRP1040138.

### Statistical analysis

Experimental results are shown as the mean of triplicate measurements. The data were represented as mean ± standard deviation using SPSS version 18.0 for Windows (SPSS Inc., Chicago, IL, USA). Differences were considered statistically significant if *P* < 0.05. Diversity index data were analyzed statistically using analysis of variance (ANOVA). Principal Coordinates Analysis (PCoA) was conducted using the QIIME package v1.8 (Caporaso et al. 2010).

## Results

### Growth performance

The effects of UA and FA on the growth performance of broiler chickens are presented in Table 3. The ADFI of F group broiler chickens were higher than those of C and U groups from 1 to 21 days, but lower than those of C group chickens from 22 to 42 days (*P *< 0.05). The ADG of F and C group broiler chickens were higher than those of U group broiler chickens from 22 to 42 days (*P *< 0.05), with no significant differences from 1 to 21 days (*P *> 0.05). The F/G ratio was lower in F and U groups broiler chickens than in C group broiler chickens from 22 to 42 days, with significant differences from days 1 through 22 (*P *< 0.05).

**Table 3:** Effects of fermented *Astragalus* on growth performance of broiler chickens

Item	Time period (days)	Dietary treatment			SEM^2^	*P*-values
		C group	U group	F group		
ADFI (g/days)	1–21	30.20 ± 2.26^a^	25.10 ± 0.22^a^	38.37 ± 1.21^b^	1.048	0.0001
	22–42	67.84 ± 9.32^a^	59.21 ± 1.11^a^	64.71 ± 0.69	3.843	0.224
ADG (g/days)	1–21	12.45 ± 0.73	12.91 ± 0.17	13.37 ± 0.50	0.369	0.178
	22–42	31.26 ± 2.22^ab^	29.79 ± 1.40^a^	34.58 ± 1.38^b^	1.209	0.035
F/G	1–21	2.43 ± 0.10^b^	1.94 ± 0.04^a^	2.87 ± 0.20^c^	0.045	0.000004
	22–42	2.16 ± 0.16^b^	1.99 ± 0.13^ab^	1.87 ± 0.55^a^	0.084	0.066

C group = basal diet; U group = basal diet + 0.5% unfermented *Astragalus*; F group=basal diet + 0.5% fermented *Astragalus*

*ADFI* average daily feed intake, *ADG* average daily gain, *F/G* feed gain ratio

^a,b,c^Means in the same row bearing different superscripts differ significantly (*P* ≤ 0.05)

### Serum biochemical parameters

The results of serum biochemical analyses are presented in Table 4. GSH-Px, AOC, and SOD were significantly higher in F group broiler chickens than in C and U group broiler chickens from 1 to 42 days (*P *< 0.05). MDA levels were lower in U group broiler chickens than in C and F group broiler chickens from 1 to 42 days (*P *<0.05). These results indicate that FA supplementation increase AOC and reduce MDA in serum.

**Table 4:** Effects of fermented *Astragalus* on the serum biochemical parameters of broiler chickens

Item	Time period (days)	Dietary treatment			SEM^2^	*P*-values
		C group	U group	F group		
CAT (U/mL)	1–21	2.26 ± 0.32	2.49 ± 0.22	2.65 ± 0.24	0.186	0.264
	22–42	5.72 ± 0.45	5.82 ± 0.52	6.01 ± 0.35	0.315	0.721
GSH-Px (U/mL)	1–21	1 127.61 ± 79.64^a^	1133.50 ± 74.79^a^	1374.21 ± 89.65^b^	57.695	0.016
	22–42	1 329.20 ± 96.00^a^	1 378.68 ± 86.00^a^	1569.12 ± 91.77^b^	64.592	0.040
AOC (U/mL)	1–21	8.90 ± 1.09^a^	9.56 ± 1.31^a^	13.23 ± 0.80^b^	0.767	0.006
	22–42	10.42 ± 0.77^a^	11.03 ± 1.25^a^	13.44 ± 1.33^b^	0.809	0.039
MDA (nmol/mL)	1–21	10.79 ± 1.27^b^	9.46 ± 1.10^b^	7.05 ± 0.85^a^	0.769	0.015
	22–42	4.23 ± 0.66	4.21 ± 0.53	3.89 ± 0.46^a^	0.395	0.713
SOD (U/mL)	1–21	118.03 ± 10.26^a^	121.45 ± 13.94^a^	156.97 ± 12.12^b^	8.627	0.014
	22–42	208.67 ± 14.06^a^	213.96 ± 16.32^a^	248.76 ± 11.85^b^	10.036	0.027

C group = basal diet; U group = basal diet + 0.5% unfermented *Astragalus*; F group=basal diet + 0.5% fermented *Astragalus*

*CAT* catalase, *GSH-Px* glutathione superoxide dismutase, *AOC* antioxidant capacity, *MDA* malondialdehyde, *SOD* suproxide dismutase

^a,b^Means in the same row bearing different superscripts differ significantly (*P* ≤ 0.05)

### Data acquisition and analysis

16S rRNA gene sequencing was performed for 45 fecal samples, resulting in 2,923,539 high-quality sequences, with an average of 64,968 reads per sample. A total of 7918 OTUs were detected based on a minimum nucleotide sequence identity of 97% for OTU clustering (Table 5). Per-sample microbial complexity was estimated with alpha-diversity indices (Chao1 and abundance-based coverage estimator (ACE) indices, Shannon and Simpson indices). The Chao1 index was used to estimate species richness and the ACE index was used to indicate the number of OTUs of the community. As shown in Table 5, the Chao1 and ACE indices were highest in the U group on day 35, followed by the C group on day 35, the C group on day 21, the F group on day 35, the U group on day 7, the U group on day 21, the C group on day 7, the F group on day 7, and the F group on day 21.The Shannon diversity index was used to evaluate the richness and evenness of the community and the Simpson diversity index was used to indicate community diversity. As shown in Table 5, the Shannon and Simpson index were highest in the C group on day 7, followed by the U group on day 21. However, the remaining groups were fairly similar. This result illustrates that fecal microbiota abundance and diversity differ by growth period.

**Table 5:** Fecal microbiota diversity estimates (N = 45)

Samples	Numbers of OTUs	Chao 1 index	ACE	Simpson index	Shannon index
C1 (7 days)	903	345.2 ± 40.92^b^	372.21 ± 55.45^b^	0.94 ± 0.04^b^	5.33 ± 0.39^b^
U1 (7 days)	946	359.6 ± 49.16^b^	394.66 ± 53.43^b^	0.90 ± 0.09^b^	5.20 ± 1.02^b^
F1 (7 days)	1162	251.8 ± 78.50^a^	277.40 ± 79.91^a^	0.71 ± 0.13^a^	3.17 ± 0.83^a^
C2 (21 days)	315	378 ± 115.16^b^	422.77 ± 117.45^b^	0.81 ± 0.13	3.95 ± 1.40
U2 (21 days)	1094	350 ± 89.34^ab^	391.33 ± 96.54^ab^	0.79 ± 0.07	3.62 ± 0.76
F2 (21 days)	1099	234 ± 75.64^a^	266.37 ± 83.81^a^	0.76 ± 0.03	2.91 ± 0.26
C3 (35 days)	1036	395.2 ± 114.48	451.65 ± 110.94	0.83 ± 0.09	3.95 ± 1.50
U3 (35 days)	1137	518.2 ± 82.16^ab^	555.51 ± 73.39^ab^	0.82 ± 0.20	4.91 ± 1.68
F3 (35 days)	1126	373.4 ± 155.75	417.11 ± 151.05	0.79 ± 0.12	3.68 ± 1.66

Means within the same column with different lowercase letters indicate significant differences (*P* ≤ 0.05). Means within the same column with the same lowercase letter or no letter indicate that the differences are not significant (*P* ≥ 0.05)

*C1* samples of group C on day 7, *C2* samples of group C on day 21, *C3* samples of group C on day 35, *U1* samples of group U on day 7, *U2* samples of group U on day 21, *U3* samples of group U on day 35, *F1* samples of group F on day 7, *F2* samples of group F on day 21, *F3* samples of group F on day 35

### Microbial beta diversity analysis

**Fig. 1:**
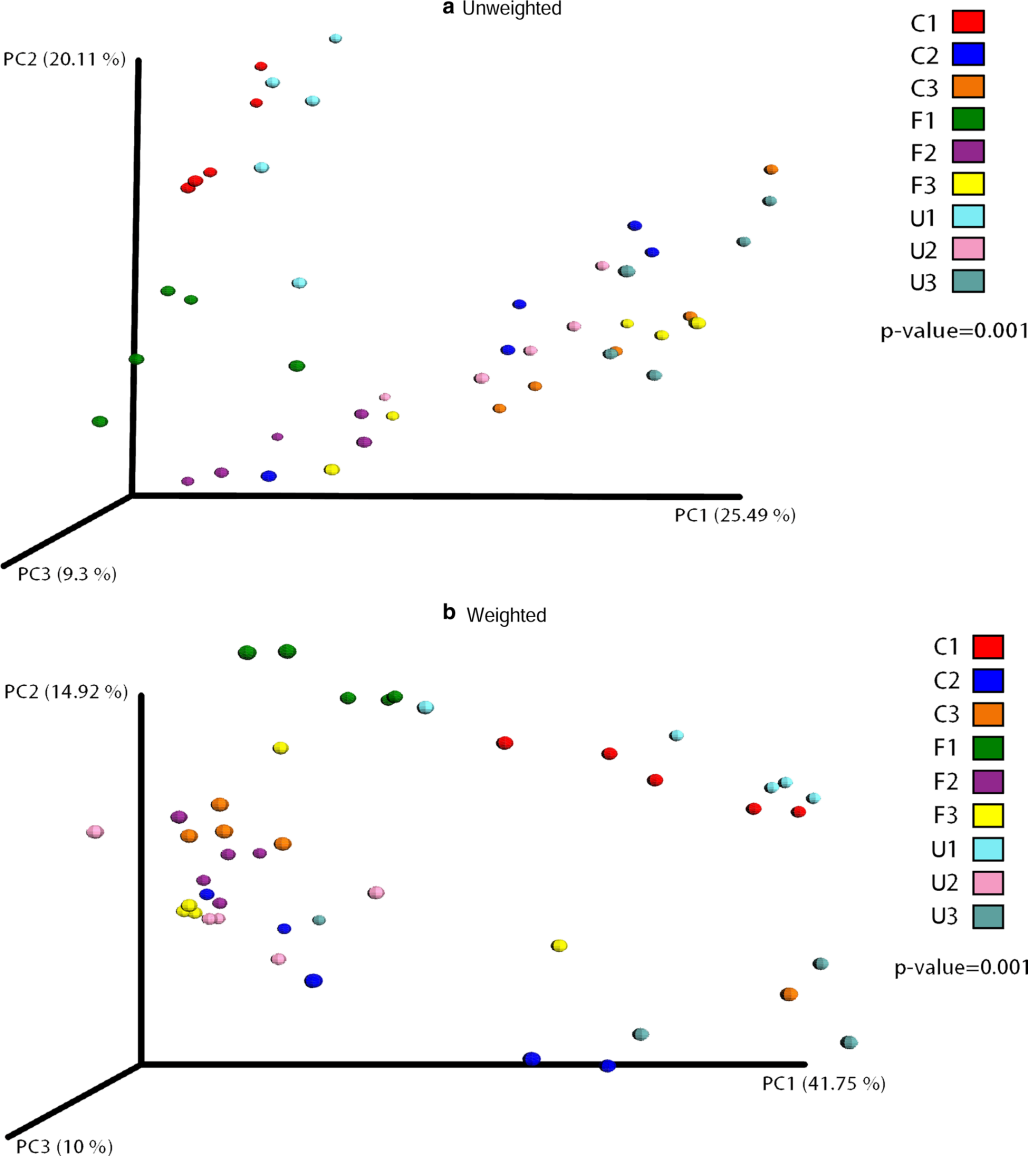
Beta diversity analysis of the 45 fecal samples. **a** Unweighted and **b** weighted UniFrac PCoA of individual chickens in each group. Individual samples are represented as group C (C1 on day 7, C2 on day 21, and C3 on day 35), group U (U1 on day 7, U2 on day 21, and U3 on day 35), and group F (F1 on day 7, F2 on day 21, and F3 on day 35)

Beta diversity analysis for the 45 fecal samples are presented by PCoA of unweighted and weighted UniFrac distance matrices (Fig. 1a, b). For the unweighted UniFrac PCoA principal component PC1, PC2 and PC3 explained 25.49%, 20.11%, and 9.3% of the between-sample variation, respectively (*P* = 0.001); for unweighted UniFrac PCoA PC1, PC2 and PC3 explained 41.75%, 14.92%, and 10% of the between-sample variation, respectively (*P* = 0.001). Both weighted and unweighted UniFrac PCoAs demonstrate a separation of all samples taken on day 7, irrespective of group. However, there was no clear separation by *Astragalus* supplementation (either fermented or unfermented) status from samples collected on days 21 and 35.

### Fecal microbiota profiles

**Fig. 2:**
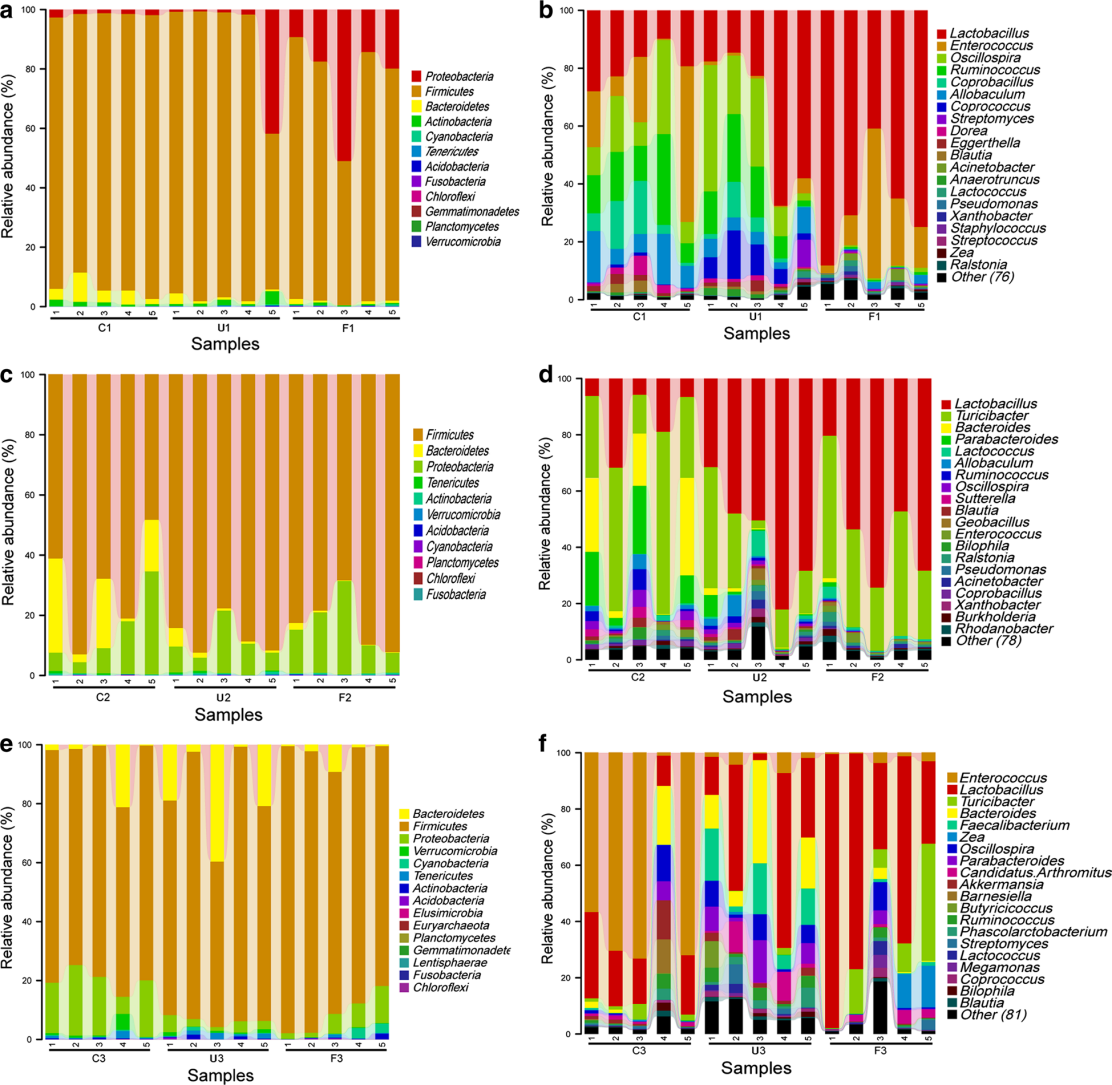
Phylum- and genus-level analysis of the 45 samples. **a** Overall fecal microbiota composition of samples at the phylum level on day 7. **b** Overall fecal microbiota composition of samples at the genus level on day 7. **c** Overall fecal microbiota composition of samples at the phylum level on day 21. **d** Overall fecal microbiota composition of samples at the genus level on day 21. **e** Overall fecal microbiota composition of samples at the phylum level on day 35. **f** Overall fecal microbiota composition of samples at the genus level on day 35

As shown in Fig. 2a, c, and e, a total of fifteen phyla were identified. *Firmicutes* was the most dominant phylum (79.1% in C, 83.9% in U and 82.2% in F), followed by *Proteobacteria* (11.1% in C, 7.5% in U and 15% in F) and *Bacteroidetes* (8.2% in C, 6.5% in U and 1.3% in F) (*P *< 0.05). On day 7 the abundance of *Proteobacteria* was significantly higher in the U and F groups than in the C group, yet significantly lower on day 35 (*P *< 0.05). The phyla *Elusimicrobia*, *Euryarchaeota* and *Lentisphaerae* were only detected on day 35 in fecal samples.

At genus level (Fig. 2b, d, and f) *Lactobacillus*, *Enterococcus*, *Turicibacter* and *Bacteroides* were the most abundant genera among the 45 samples. On day 7 *Lactobacillus* was present at 19.6%, 38.2% and 69.8% in C, U and F groups (*P *< 0.05), respectively; on day 21 *Lactobacillus* was present at 10.8%, 52.8% and 52.4% in C, U and F groups (*P *< 0.05), respectively; and on day 35 *Lactobacillus* was present at 19.2%, 28.4% and 56.6% in C, U and F groups (*P *< 0.05), respectively. This indicates that *Lactobacillus* was consistently higher in U and F groups than in the C group. On day 35, *Enterococcus* abundance was lower in the U and F groups than in the C group. The C group was dominated by *Enterococcus* at 54.4%. *Turicibacter* was higher on day 21 than on day 7 and 35 in the F group. The F group was dominated by *Turicibacter* on day 21 (33.5%). *Bacteroides* was highest in the U group on day 35 (17.8%), follow in the C group on day 21 and 35. other groups was lower.

### Community composition heatmap and cluster analysis

**Fig. 3:**
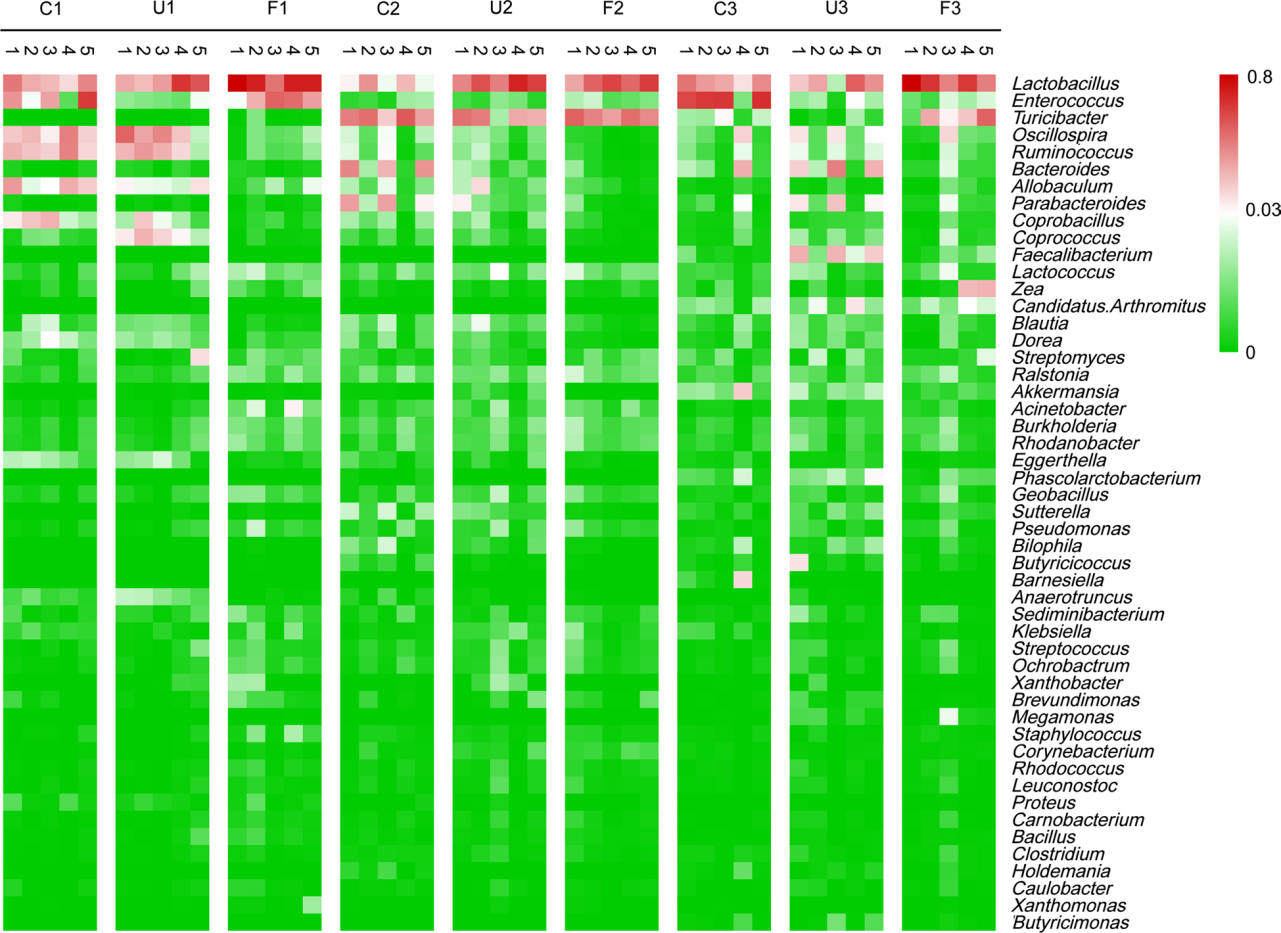
Heatmap of the top 50 most abundant genera summarized by group. The top 50 most abundant genera were clustered using R software. The genera with higher abundances in the corresponding samples are shown in red, while those with lower abundances are shown in green. *Lactobacillus* was the most abundant genus on day 7 in F group. *Enterococcus* were more abundant in group C than in groups U and F on day 35

As shown in Fig. 3, the top 50 most abundant genera were clustered and presented as a heatmap. *Lactobacillus* was the most abundant genus in all samples at all three time points, irrespective of supplementation with UA or FA. Moreover, its abundance was higher in the U and F groups than in the C group on days 7, 21 and had the highest abundance in F groups on days 7. The C and U groups had higher abundances of *Oscillospira* and *Ruminococcus* than the F group on day 7. *Enterococcus* was more abundant in group C than in groups U and F. Interestingly, *Turicibacter* was more abundant on day 21 in the C, U, and F groups, although its abundance was lower on days 7 and 35.

In addition, the abundances of *Allobaculum* and *Coprobacillus* were higher in the C and U group on day 7, while the abundance of *Coprococcus* was higher only in the U group on day 7. *Faecalibacterium* had higher abundance in the U group on day 35. These results indicate that both unfermented and fermented dietary *Astragalus* alters the relative abundance of certain genera in the fecal microbiota of broiler chickens.

## Discussion

It is becoming increasingly apparent that gut microbiota play an important role in maintaining host health (Yeoman et al. 2012). However, very few studies have assessed the interaction between fermented *Astragalus* and fecal microbiota of broiler chickens. The present study was designed to assess the potential of FA as a feed additive by evaluating its effects on growth performance, serum biochemical parameters, and fecal microbiota of broiler chickens. *Astragalus* is rich in polysaccharides, saponins, and flavonoids, which makes it amenable to fermentation by *Aspergillus oryzae* M29, which increases its bioactive substance content through bioconversion (Sheih et al. 2011). In this study we show that *Astragalus* fermentation with *L. plantarum* significantly increased the production of polysaccharides and organic acids. This may be due to degradation of cell wall cellulose by digestive enzymes, allowing the release of more polysaccharides and extracellular polysaccharides during the fermentation process (Timmerman et al. 2014).

Previous studies have reported that certain fermented herb additives, such as *Ginkgo biloba* leaves, *red ginseng*, and *water*-*plantain* improve growth performance and feed efficiency in livestock (Ao et al. 2011; Cao et al. 2012; Hossain and Yang 2014). In the present study, these parameters were evaluated in chickens fed diets supplemented with FA. We show significantly higher growth performance and lower F/G ratios relative to chickens fed UA or control diets. The reason for these improvements may be that fermentation improves the flavor and palatability of feed and increases the activity of digestive enzymes in the intestinal tracts of livestock (Czech et al. 2009). Such plant-based water-soluble polysaccharides could enhance intestinal function in a manner similar to prebiotics (Li et al. 2009).

During fermentation of *Astragalus* with *L. plantarum,* a large amount of organic acids are produced, including acetic acid, methylacetic acid, ethyl acetic acid and lactic acid. These organic acids could decrease the pH of fermented *Astragalus* (Qiao et al. 2018b), which may inhibit pathogenic bacteria and maintain the balance of the intestinal microbiota. Simultaneously, the results of this study suggest increased *Astragalus* polysaccharides yield using *L. plantarum* fermentation. This may promote gut microbial fermentation of herbal carbohydrates yielding short-chain fatty acids (*n*-butyrate, acetate, and propionate), which are either absorbed across the gut epithelium into the circulation or utilized by enterocytes and can have numerous host beneficial effects (Xu et al. 2017). The SCFA butyrate has been shown to improve growth performance (Panda et al. 2009).

Our results suggest that diets supplemented with FA positively affect serum biochemical parameters in broiler chickens. The antioxidant enzymes SOD, GSH-Px, and AOC play an important role in balancing redox status (Wu et al. 2015). In this study serum SOD, GSH-Px, and AOC were higher in broiler chickens fed FA, which is in agreement with a previous study demonstrating improved antioxidant status following addition of *Astragalus* powder to the feed of broiler chickens (Zhang et al. (2013). One possible explanation for this is that SOD and GSH-Px have antioxidant activity and protect cells from free radicals (Fridovich 1995); a second possibility is that lactic acid bacteria (LAB), which are widely used in fermentation, also have antioxidant properties (Liu and Pan 2010; Wang et al. 2009). In addition, we showed that the production of polysaccharides and flavonoids by *Astragalus* was increased by fermentation with LAB. Antioxidant properties may also be affected by polysaccharides, phenolics, and flavonoids (Wu et al. 2015). It has been reported that polysaccharides have antioxidant potential and can promote serum and hepatic antioxidant enzyme activity in chickens and rats (Deng and Hu 2011; Saleh et al. 2013; Sun and Wang. 2010; Yan et al. 2010).

We analyzed the fecal microbiota of broiler chickens fed with UA and FA. The alpha-diversity analyses showed that the three groups exhibited greater species richness and diversity on day 35 and U groups was highest, as indicated by the Chao 1 and ACE. Previous results suggest that the intestinal microbiota population of chickens becomes more diverse with aging (Shaufi et al. 2015), which is in agreement with our results. We report differences in beta-diversity between microbial communities of the C, U, and F groups on day 7. These results may be explained by the UA and FA feed additives, which had different pH values, contained the probiotic *L. plantarum* in FA, and microbial secondary metabolites that may have an effect on the fecal microbiota.

At the phylum-level, *Firmicutes* was the most dominant phylum, followed by *Proteobacteria* and *Bacteroidetes*, in all three groups on days 7, 21, and 35. In the F group on day 35, *Firmicutes* accounted for more than 90% of all bacterial sequences. These results are consistent with previous reports, where *Firmicutes* was the most dominant taxon in broiler chickens (Xu et al. 2016; Danzeisen et al. 2011; Qu et al. 2008; Yeoman et al. 2012). These results could be partially attributed to the effect of fermented *Astragalus* on some bacteria. It is believed that in the F group, fermented *Astragalus* played a role in the degradation of plant polysaccharides, promoted nutrient digestion and absorption, and maintained fecal microbial diversity. Future studies are needed to ascertain the underlying cause of these results.

At the genus-level, *Lactobacillus* had the highest abundance in F group on day 7 (Figs. 2, 3), which is in agreement with a previous report (Ding et al. 2017). However, the U and F groups had a higher proportion of *Lactobacillus* relative to controls (C group) across all three time points. Importantly, a potentially pathogenic genus *Enterococcus* (two species were common: *E. faecalis* and *E. faecium*) abundance was lower in the U and F groups than in the C group on day 35 (*P* < 0.05), which indicates that dietary supplementation with UA and FA effectively modulated the fecal microbiota in the later stage of broiler chickens. This may be the reason for later promotion of weight gain and antioxidant status in U and F groups relative to controls.

In this study, fermenting *Astragalus* resulted in an enrichment in polysaccharides, flavonoids, saponins and organic acids. The polysaccharide content may be influenced by intestinal microbiota and activate to generate enzymes resulting in higher abundance of *Lactobacillus* in chickens fed fermented *Astragalus*. *Lactobacillus* can complement other beneficial microbes to increase the efficiency of feed utilization in broiler chickens (Saito et al. 2003). Moreover, it is well documented that flavonoids maintain intestinal barrier integrity, modulate the secretion of gut hormones and shape microbiota composition and function (Oteiza et al. 2018). In addition, gut microbiota can hydrolyze absorbed saponins that may generate potential health benefits (Lim et al. 2015). Therefore, broiler chicken growth was improved in chickens receiving dietary fermented *Astragalus* due to an increase in available polysaccharides, flavonoids, saponins, and microbially-generated organic compounds.

Our results indicate that FA-supplemented diets improve growth performance and serum biochemical parameters in broiler chickens, and that both UA and FA modulate the fecal microbiota of broiler chickens. This is the first study to show that unfermented and fermented *Astragalus* affect fecal microbiota in broiler chickens. Recent studies have reported that gut microbiota can transform herbs into metabolites, which improve the composition of gut microbiota (Xu et al. 2017). Although the effects of herbs have been investigated on gut microbial populations of chickens and humans, the exact mechanisms remain unclear. We speculate that UA and FA alter fecal microbiota diversity because of their anti-inflammatory and antioxidative properties (Kim et al. 2013), which can adjust the immune balance and potentially reduce the need for antibiotics in chicken feed (Sanpha et al. 2013). Moreover, *Astragalus* is fermented by *L. plantarum* to produce large amounts of organic acids, such as lactic acid, methyl acetic acid, acetic acid, and ethyl acetic acid, which may be advantageous to the population of beneficial bacteria, while inhibiting the propagation of certain harmful bacteria, although further investigation is needed in this regard.

Despite their preliminary nature, our results indicate that the composition of fecal microbiota of broiler chickens fed UA and FA differed from that of broiler chickens fed a standard diet. This study provides a foundation for further studies on the interaction between microbiota and *Astragalus*.

## Abbreviations

HMs: herbal medicines
FA: fermented *Astragalus*
SSF: solid state fermentation
CGMCC: China General Microbiological Culture Collection Center
MRS: de Man, Rogosa, and Sharpe
CFU: colony-forming units
BW: body weight
ADFI: average daily feed intake
ADG: average daily gain
F/G: feed to gain ratio
CAT: levels of serum catalase
GSH-Px: glutathione superoxide dismutase
MDA: malondialdehyde
SOD: superoxide dismutase
AOC: antioxidant capacity
RDP: Ribosomal Database Project
NGS: high-throughput, next-generation sequencing
OTUs: operational taxonomic units
PCR: polymerase chain reaction
PCoA: principal coordinate analysis
UA: unfermented *Astragalus*
ACE: abundance-based coverage estimator
LAB: lactic acid bacteria
